# Macrophage Dectin-1 mediates Ang II renal injury through neutrophil migration and TGF-β1 secretion

**DOI:** 10.1007/s00018-023-04826-4

**Published:** 2023-06-20

**Authors:** Shiju Ye, He Huang, Yun Xiao, Xue Han, Fengjie Shi, Wu Luo, Jiawen Chen, Yang Ye, Xia Zhao, Weijian Huang, Yi Wang, Dongwu Lai, Guang Liang, Guosheng Fu

**Affiliations:** 1 Key Laboratory of Cardiovascular Intervention and Regenerative Medicine of Zhejiang Province, Department of Cardiology, Sir Run Run Shaw Hospital, School of Medicine, Zhejiang University, Hangzhou, 310020 Zhejiang China; 2 School of Pharmaceutical Sciences, Hangzhou Medical College, Hangzhou, 311399 Zhejiang China; 3 Chemical Biology Research Center, School of Pharmaceutical Sciences, Wenzhou Medical University, Wenzhou, 325035 Zhejiang China; 4 Department of Cardiology, The First Affiliated Hospital of Wenzhou Medical University, Wenzhou, 325035 Zhejiang China

**Keywords:** Hypertension, Dectin-1, Angiotensin II, Chronic kidney disease, TGF-β1

## Abstract

Macrophage activation has been shown to play an essential role in renal fibrosis and dysfunction in hypertensive chronic kidney disease. Dectin-1 is a pattern recognition receptor that is also involved in chronic noninfectious diseases through immune activation. However, the role of Dectin-1 in Ang II-induced renal failure is still unknown. In this study, we found that Dectin-1 expression on CD68 + macrophages was significantly elevated in the kidney after Ang II infusion. We assessed the effect of Dectin-1 on hypertensive renal injury using Dectin-1-deficient mice infused by Angiotensin II (Ang II) at 1000 ng/kg/min for 4 weeks. Ang II-induced renal dysfunction, interstitial fibrosis, and immune activation were significantly attenuated in Dectin-1-deficient mice. A Dectin-1 neutralizing antibody and Syk inhibitor (R406) were used to examine the effect and mechanism of Dectin-1/Syk signaling axle on cytokine secretion and renal fibrosis in culturing cells. Blocking Dectin-1 or inhibiting Syk significantly reduced the expression and secretion of chemokines in RAW264.7 macrophages. The in vitro data showed that the increase in TGF-β1 in macrophages enhanced the binding of P65 and its target promotor via the Ang II-induced Dectin-1/Syk pathway. Secreted TGF-β1 caused renal fibrosis in kidney cells through Smad3 activation. Thus, macrophage Dectin-1 may be involved in the activation of neutrophil migration and TGF-β1 secretion, thereby promoting kidney fibrosis and dysfunction.

**Graphical Abstract:**

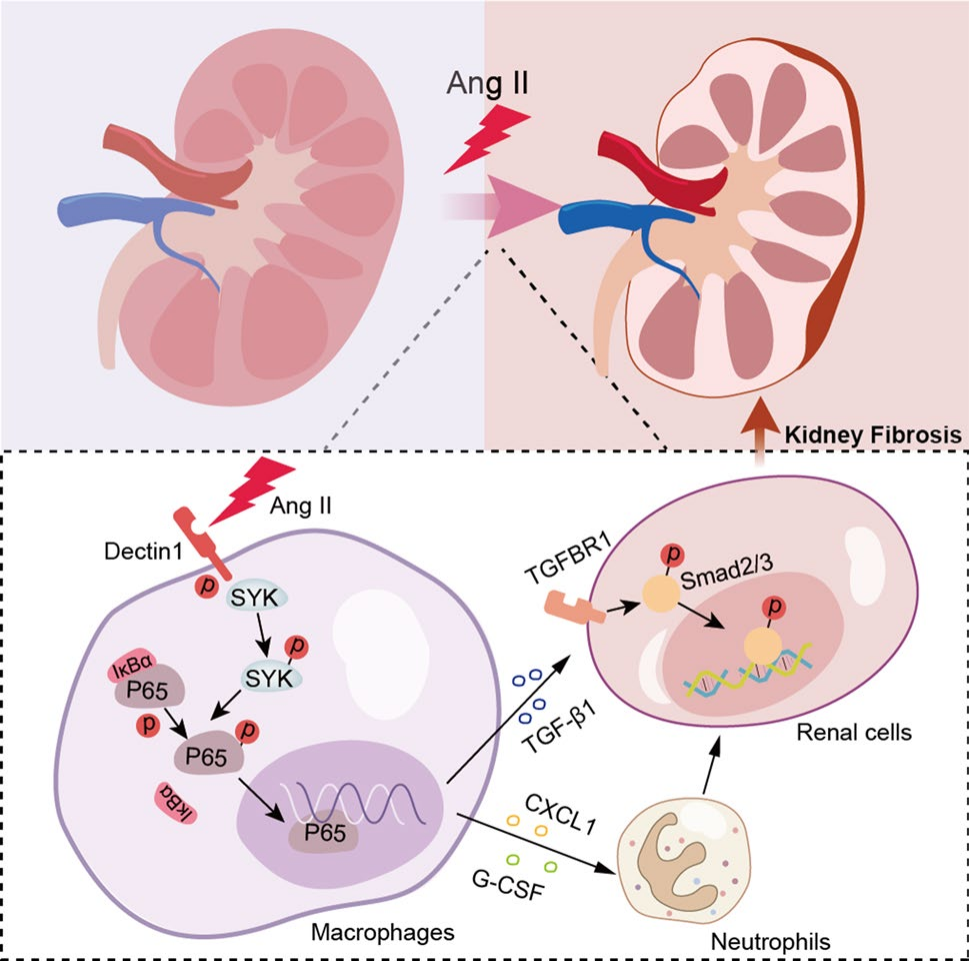

**Supplementary Information:**

The online version contains supplementary material available at 10.1007/s00018-023-04826-4.

## Introduction

Hypertension is the leading cause of secondary chronic kidney disease (CKD) [1]. Elevated Ang II levels are the most powerful mediators of the development of hypertension [2–4]. Currently, several drugs used for hypertension, including angiotensin-converting enzyme (ACE) inhibitors and angiotensin II receptor blockers (ARBs). However, these blood pressure-controlling drugs could not completely block the progression of hypertensive complications including CKD. Thus, targeting new pathogenic mechanism of Ang II-related hypertensive renal complication is needed. The inflammatory response and innate immune activation are important pathogenic mechanisms associated with Ang II [5–7]. Prolonged and continuous low-grade inflammation induced by Ang II ultimately results in the development of renal fibrosis and end-stage renal disease [8]. In addition, accumulating evidence has demonstrated the crucial role of immune cells, especially infiltrated macrophages, in renal injury secondary to hypertension. Thus, it is vital to examine the mechanism by which these immune cells are attracted to the kidney and cause kidney fibrosis. Accumulating evidence has shown the involvement of the innate immune system in noninfectious diseases, such as atherosclerosis, acute kidney injury, hypertensive kidney disease [9], and other types of CKD [10, 11]. During CKD, sustained activation of proinflammatory monocytes and macrophages leads to glomerulosclerosis and renal interstitial fibrosis, which accelerates renal failure and precedes the onset of end-stage renal disease [12]. Neutrophils accumulate in sites of interstitial renal fibrosis after Ang II infusion [6]. Accordingly, therapeutic targeting of these immune cells and key proteins may attenuate hypertensive kidney disease induced by Ang II.

Dectin-1, a C‐type lectin receptor (CLR) belonging to the family of pattern recognition receptors (PRRs), was shown to recognize β-glucan during microbial infections and trigger the innate immune response [13]. Upon activation by ligands, Dectin-1 recruits the directly downstream spleen tyrosine kinase (Syk) to induced Syk phosphorylation, which subsequently activates nuclear factor-κB (NF-κB) to trigger the gene expression of cytokines and chemokines, including tumor necrosis factor-α (TNF-α), interleukins (ILs), and CXC-chemokine ligand 1 (CXCL1). Recent studies have also identified several endogenous ligands of Dectin-1, such as Galectin-9 in an experimental autoimmune encephalomyelitis (EAE) model [14] and in pancreatic carcinoma [15], Annexins in autoimmune diseases and aging [16], and Vimentin in atherosclerosis [17]. Thus, Dectin-1 also plays a crucial role in non-infectious inflammatory diseases through its recruitment and activation of immune cells.

In kidney, Dectin-1 has been reported to be involved in the renal immune response to fungal infection. Macrophage Dectin-1 recognizes β-glucan, leading to the activation of PKCδ, which enhances neutrophil metabolic fitness in renal responses to *Candida albicans* [18]. In addition, Dectin-1 activation also increased systemic inflammation in *Candida*-associated renal ischemia/reperfusion (I/R) injury [19], and IFN-β production induced by renal dendritic cell-derived Dectin-1 is crucial for defense against *C. albicans* infection [20]. Collectively, Dectin-1 in macrophages robustly actives the immune response in infectious renal diseases and has proinflammatory effects on noninfectious diseases. However, whether Dectin-1 plays a role in non-infectious kidney diseases, including Ang II/hypertension-induced CKD, remains unknown.

In this study, we examined the expression of Dectin-1 in Ang II infusion-induced renal injury and identified the underlying mechanism by which Dectin-1 mediates hypertensive renal fibrosis and kidney failure. We demonstrated that macrophage-derived Dectin-1/Syk plays a powerful pathogenic role in renal fibrosis through the activation of neutrophil migration and TGF-β1 secretion.

## Methods

### Reagents

Ang II (HY-1394) was purchased from MedChemExpress (Shanghai, China). Primary antibodies against Syk (2715S), NF-κB p65 (8242 T), Ly-6G (FITC Conjugate) (88876S), P-Smad3 (9520), Lamin B1 (13435) and Normal Rabbit IgG (2729) were purchased from Cell Signaling Technology (Beverly, MA, USA). Antibodies against Dectin-1 (ab140039), P-Syk (phospho Y323) (ab62338), and G-CSF (ab181053) were purchased from Abcam (Shanghai, China). Antibodies against AQP1 (sc-25287) and Wt1 (sc-7385) antibodies were obtained from Santa Cruz Biotech (Texas, USA). Antibodies against CXCL1 (12335–1-AP), CXCR2 (20634-1-AP), Collagen Type I (67288–1-Ig), Collagen Type IV (55131–1-AP), TGF-β1(21898-1-AP), α-SMA (14395–1-ap), Smad3 (66516–1-Ig), and GAPDH (60004-1-Ig) were purchased from Proteintech (Shanghai, China). Antibodies against CD68 (MA5-13324) were purchased from Thermofisher Scientific (Waltham, MA, USA). Neutralizing monoclonal antibody against mouse Dectin-1 (R1-8g7) were obtained from InvivoGen (San Diego, CA, USA). R406 (S2194) was purchased from Sellerk (Shanghai, China). Secondary anti-rabbit IgG HRP (7074), anti-mouse IgG HRP (7076) and anti-rat IgG HRP (7077) were purchased from Cell Signaling Technology (Beverly, MA, USA). Goat anti-Mouse IgG Secondary Antibody/Alexa Fluor 488 (A11001) and Donkey anti-Rabbit IgG Secondary Antibody/Alexa Fluor 568 (A10042) were purchased from ThermoFisher (Waltham, MA, USA).

### In vivo randomization and blinding procedures

The random number table was used for randomization, as previously described [21]. All animal experiments were performed and analyzed in a blinded manner. Animals were assigned to the Ctrl and Ang II groups in a randomized fashion. When the mice were randomly divided into groups, they were given their permanent numerical designations in the cages. For each group, one cage was selected randomly from the pool of all cages. All data were collected and analyzed by two observers who were blinded to the group assignments and animal treatments.

### Ang II-induced kidney injury in mice

All animal care and experimental procedures were approved by the Zhejiang Chinese Medical University Laboratory Animal Research Center and Welfare Committee (20201116-06), and all animals received humane care according to the National Institutes of Health (USA) guidelines. The C57BL/6 male mice used in this study were obtained from Zhejiang Chinese Medical University Laboratory Animal Research Center and Clec7a-KO mice (Dectin-1^−/−^ mice, D1KO mice, Strain NO.T011442) were purchased from GemPharmatech (Nanjing, China). The mice were housed with a 12:12 h light–dark cycle and a constant room temperature and were fed a standard rodent diet. The mice were acclimatized to the laboratory for at least 2 weeks before initiating the studies. Detailed methods for these models are described below.

Eight-week-old C57BL/6 mice (wild-type, WT mice) and Dectin-1^−/−^ mice (D1KO mice) were randomly divided into four groups: (i) WT controls (WT-Ctrl group, n = 7); (ii) Ang II-infused WT mice (WT-Ang II group; n = 7); (iii) D1KO controls (WT-D1KO group; n = 7); and (iv) Ang II-infused D1KO mice (WT- D1KO group; n = 7). Ang II was administered using osmotic mini-pumps that delivered 1000 ng/kg/min Ang II (Alzet MODEL 1004, CA, USA) for 4 weeks, as described previously [22–24].

At the indicated time points, systolic blood pressure was measured by non-invasive tail-cuff Pressure Analysis System while mice were conscious (BP-98A; Softron, Tokyo, Japan). Specifically, each mouse was fixed on a constant temperature table at 37 ℃ and rested quietly for 10 min before blood pressure was measured with a tail-cuff mounted at the tail 0.5 cm from the rump of the mouse, and each mouse was measured three times consecutively and the average value was taken. Measurements were performed during 1:00 pm to 5:00 pm with 5 days of previous training. When animals were sacrificed using sodium pentobarbital anesthesia. Kidney tissues were snap-frozen in liquid nitrogen for gene and protein expression analyses or fixed with 4% paraformaldehyde for histological analysis.

### Kidney functional tests

The level of BUN and Creatinine in serum were measured followed the instructions of BUN assay kit (C013-2-1, Nanjing Jiancheng, China) and Creatinine assay kit (C011-2-1, Nanjing Jiancheng, China).

### Kidney tissue staining

The kidneys were fixed in 4% paraformaldehyde and embedded in paraffin. 5 μm thick sections were stained with hematoxylin and eosin (H&E) (Solarbio Life Sciences, Beijing, China) for routine histological analysis and Sirius red and Masson’s Trichrome (Solarbio Life Sciences, Beijing, China) to evaluate renal fibrosis following the manufacturer’s instructions [25].

To detect renal fibrosis, paraffin sections were also stained with 0.1% Sirius Red F3B (Solarbio Life Science, Beijing, China) and 1.3% saturated aqueous solution of picric acid or Masson’s Trichrome (Solarbio Life Sciences, Beijing, China) to evaluate collagen deposition. To quantify the level of fibrosis, 10 non‐overlapping fields in each tissue (n = 7) were scored on a semiquantitative scale (< 5%, 5–10%, 10–25%, 25–50%, 50–75%, and 75–100%), relative to total tissue area in the field. Sections were observed under a light microscope (Nikon, Japan).

For immunohistochemical staining, sections were deparaffinized and rehydration. Sections were treated with 3% H2O2 for 30 min to block endogenous peroxidase activity and then with 1% BSA in PBS for 30 min. Slides were incubated overnight at 4 °C with primary antibody (TNF-α, 1:50, cat:60291-1-Ig; F4/80, 1:50, cat: 28463-1-AP; Proteintech). Peroxidase-conjugated secondary antibodies were used for detection (ThermoFisher; 1:500 dilution; 1 h incubation). Slides were counterstained hematoxylin for 5 min, dehydrated, and mounted. Images were viewed by a bright field microscope (Nikon).

For immunofluorescence staining, 5 μm thick OCT-embedded sections were prepared from kidneys, and fixed with 4% paraformaldehyde for 10 min and then with 5% BSA in PBS for 30 min. Appropriate antibodies were used to detect the specific protein expression. Primary antibodies used were rabbit polyclonal anti-Dectin-1 (ab140039), mouse Monoclonal anti-CD68 (MA5-13324), mouse anti- AQP1 (sc-25287), mouse anti-Wt1 (sc-7385), rat anti-Ly-6G/FITC Conjugated (88876S), rabbit polyclonal anti-G-CSF (ab181053), rabbit polyclonal anti-CXCL1 (12335-1-AP), and the samples were incubated with these antibodies overnight at 4 ℃. The appropriate Alexa-Fluor-coupled secondary antibodies were used, especially in double-staining experiments, and they were applied for 1 h at room temperature. Afterward, the sections were counterstained with DAPI and coverslipped, which was followed by analysis using fluorescence microscopy with an Olympus microscope. Images were quantified in a blind manner using ImageJ (version 1.38 ×  National Institutes of Health, Bethseda, MD, USA). For each kidney sample, at least five random fields were measured.

### Transcriptome analyses

Levels of *Clec7a* in renal biopsies from healthy living donors (HL, n = 22) and patients with hypertensive nephropathy (HN, n = 15) were determined from publicly available transcriptome data. GSE37460 was selected. The *Clec7a* levels were determined using data analysis tools for expression profiling by array data or gene ID conversion tools for expression profiling by high-throughput sequencing data.

### Human subject study

In this study, archived frozen human kidney tissues were obtained from the First Affiliated Hospital of Wenzhou Medical University. The study was approved by the Ethical Committee of the First Affiliated Hospital of Wenzhou Medical University (Ethics Number: LCYJLS‐2018‐180) and completed according to the Declaration of Helsinki. Three samples were obtained from subjects with hypertension and 3 without hypertension; the subjects were matched for age and sex and obtained in a double-blind manner. These tissues were obtained at the time of nephrectomy for conventional renal carcinoma. However, nontumoral specimens were used in this study. Sufficient frozen tissue was obtained for immunofluorescence staining. The details on subjects were collected, described, and used in our previous publication *Hypertension. 2022 Sep;79(9):2028–2041* [26].

### Cell culture

The mice macrophages line RAW 264.7 cells (RAW cells) and glomerulus myofibroblast-like cell line SV40 cells were obtained from the Shanghai Institute of Biochemistry and Cell Biology (Shanghai, China).

RAW cells were cultured in Dulbecco’s Modified Eagle Medium (DMEM; Gibco/BRL life Technologies, Eggenstein, Germany) supplemented with 10% fetal bovine serum (Hyclone, Logan, UT), 100 U/mL penicillin, and 100 U/mL streptomycin. All cells were incubated in the serum-free medium for 12 h before the stimulation.

SV40 cells as the glomerulus myofibroblast-like cell line [27] were cultured in Dulbecco’s Modified Eagle Medium (DMEM; Gibco/BRL life Technologies, Eggenstein, Germany) supplemented with 10% fetal bovine serum (Hyclone, Logan, UT), 100 U/mL penicillin, and 100 U/mL streptomycin.

### Immunofluorescence staining

For NF-κB (p65) translocation, cells were fixed with 4% paraformaldehyde for 10 min, permeabilized with 0.1% Triton X-100 for 10 min and then with 5% BSA in PBS for 30 min. Next, Cells were incubated with anti-NF-κB (p65) antibody (1:200) overnight at 4 °C. Goat Anti-Rabbit IgG H&L (FITC) (1:1000) was used for detection. Immunofluorescence was viewed and captured using Olympus fluorescence microscope.

For TGF-β1 staining, cells were fixed with 4% paraformaldehyde for 10 min, permeabilized with 0.1% Triton X-100 for 10 min and then with 5% BSA in PBS for 30 min. Next, Cells were incubated with anti-TGF-β1antibody (1:200) overnight at 4 ℃. Goat Anti-Rabbit IgG H&L (TRITC) (1:1000) was used for detection. Immunofluorescence was viewed and captured using Olympus fluorescence microscope.

For Smad3 translocation, cells were fixed with 4% paraformaldehyde for 10 min, permeabilized with 0.1% Triton X-100 for 10 min and then with 5% BSA in PBS for 30 min. Next, Cells were incubated with anti-Smad3 antibody (1:200) overnight at 4 ℃. Goat Anti-mouse IgG H&L (FITC) (1:1000) was used for detection. Immunofluorescence was viewed and captured using Olympus fluorescence microscope.

### ELISA

The levels of G-CSF (MultiSciences, cat:70-EK269/2), CXCL1(MultiSciences, cat:70-EK296/2) and TGF-β1 (elabscience, cat: E-EL-M2612c) in supernatant of RAW cells were measured using the ELISA kit. All experiments followed the instructions.

### Chromatin immunoprecipitation- quantitative RCR

Chromatin immunoprecipitation (ChIP) was performed using SimpleChIP enzymatic chromatin IP kit with magnetic beads (9003, Cell Signaling Technology), with anti- NF-κB p65 (8242 T) or IgG (2729). Quantitative PCR was performed with SYBR Green (QuantStudio, Thermo Fisher). Primers for TGF-β1 promoter sites were designed using Oligo 7 (oligo.net) and NCIB Primer Design Tool, and are presented in Supplementary Table S1.

### Real-time quantitative PCR

RNA was isolated from cultured cells and kidney tissues using TRIZOL (Thermo Fisher, cat: 15596026) or RNA kit (esscience, cat: RN001). PrimeScript™ RT reagent Kit (Takara, cat: RR037A) was used for cDNA synthesis. Quantitative real-time PCR was performed using ABI QuantStudio6 detection system (Applied Biosystems, Thermo Fisher). Primers for genes were synthesized and obtained from Thermo Fisher. Sequences are presented in supplementary Table S1. mRNA of target genes was normalized to β-actin housekeeping gene.

### Western blot analysis

Fifty micrograms of cell and tissue lysates were separated by 10% SDS-PAGE and electro-transferred to a PVDF membranes. Membranes were blocked in Tris-buffered saline containing 0.05% Tween20 and 5% non-fat milk for 1.5 h. PVDF membranes were then incubated with specific primary antibodies. Immunoreactive bands were detected by incubating with secondary antibodies conjugated to horseradish peroxidase and enhanced chemiluminescence reagent (Bio-Rad). Densitometric quantification was performed using Image J analysis software version 1.38e and normalized to their respective control (GAPDH for cytosolic proteins, Lamin B for nuclear fractions, and total protein for phosphorylated-form detection).

### Statistical analysis

The data presented in this study are representative of at least 3 independent experiments and are expressed as the mean ± SEM. The exact group size (n) for each experiment is shown, and ‘n’ refers to independent values, not technical replicates. Statistical analysis was performed with GraphPad Prism 8.0 software (San Diego, CA, USA). The Shapiro–Wilk test was used to determine the normality of the data, and all data in this study passed the normality distribution test. Comparisons between two groups were analyzed by 2-tailed Student’s t test. We used one-way ANOVA followed by Tukey’s post-hoc test when comparing more than two groups of data. Additionally, for samples that required a single mouse to be measured more than once, data sets were analyzed independently using 2-way repeated-measures ANOVA with a single pooled variance and a Tukey correction for pairwise comparisons within groups for each data set. P ˂0.05 was considered statistically significant. Posttests were performed only if the F achieved P < 0.05 and there was no significant variance in homogeneity.

## Results

### Ang II infusion induced Dectin-1 expression in the kidney

**Fig. 1:**
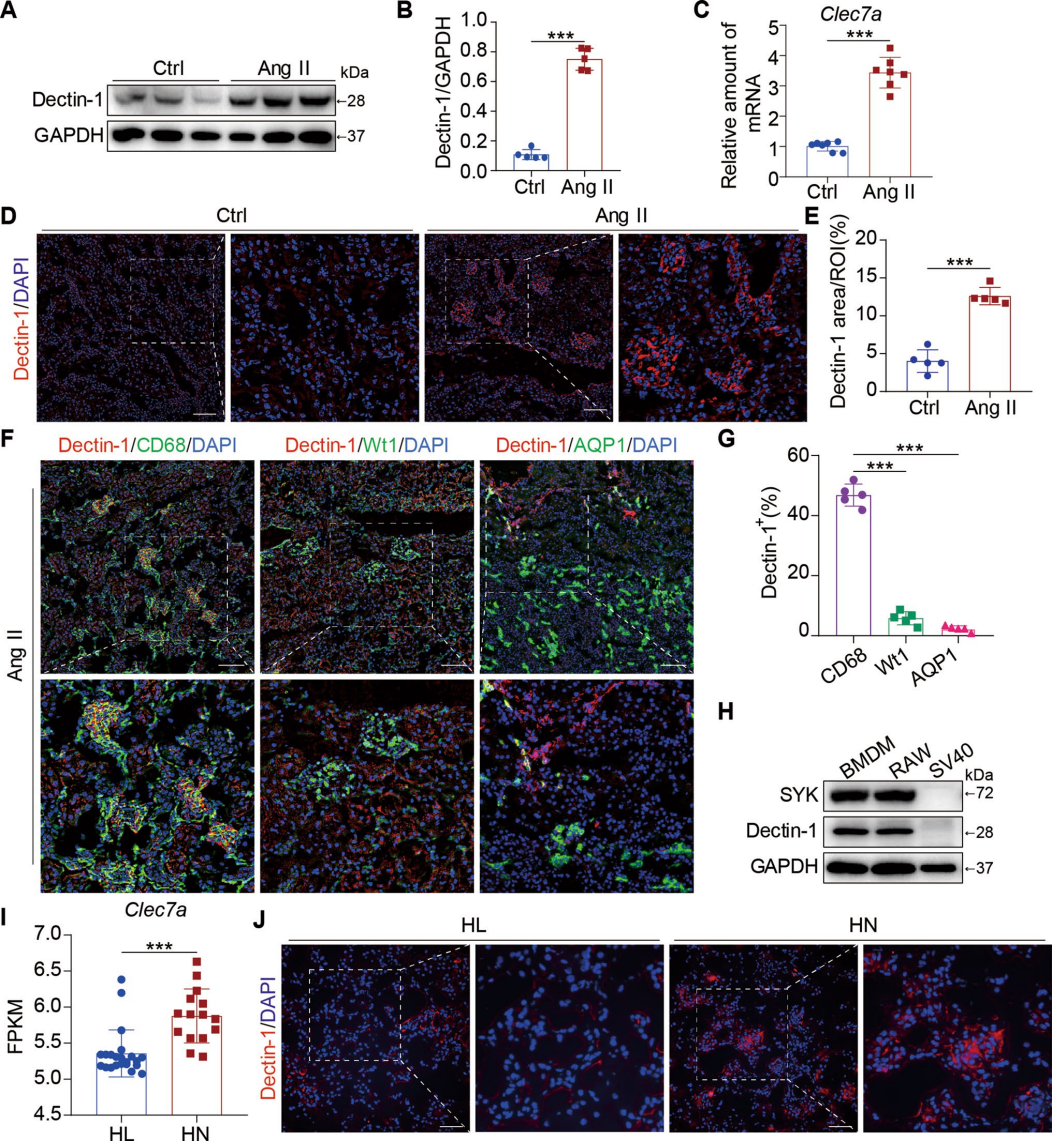
Ang II infusion induced Dectin-1 levels in the kidney. **A** Representative western blot analysis of Dectin-1 expression in kidney tissue from Ang II infusion mice and control ones. GAPDH was used as loading control; **B** Densitometric quantification of immunoblots in Fig. 1A showing Dectin-1: GAPDH; **C** Real-time qPCR showing mRNA levels of *Clec7a* in the kidney tissues; **D** Representative immunofluorescence staining of Dectin-1 in murine kidney after Ang II infusion mice and control ones (n = 5; scale bar, 50 μm); **E** Quantification of Dectin-1 positive areas (%) from immunofluorescence staining of Dectin-1; **F** Representative dual-immunofluorescence staining of Dectin-1 and CD68, Dectin-1 and Wt1 or Dectin-1 and AQP1 in murine kidney after Ang II infusion for 4 weeks (n = 5; scale bar, 50 μm); **G** Quantification of Dectin-1 and CD68, Dectin-1 and Wt1 or Dectin-1 and AQP1 positive areas (%) from dual-immunofluorescence staining (F); **H** Representative western blot analysis of the levels of SYK and Dectin-1 expression in BMDM, RAW cell lines, and SV40 cell lines; **I** The relative levels of *Clea7a* in renal biopsies from healthy living donors (HL, n = 22) and patients with hypertensive nephropathy (HN, n = 15), identified from a publicly available study: GSE37460. Data were analyzed to confirm the findings of the present study. **J** Representative immunofluorescence staining of Dectin-1 in renal tissues from patients with HN and HL (n = 3; scale bar, 50 μm). [A-G, n = 5; J, n = 3: Student’s t test; *P < 0.05, **P < 0.01, and ***P < 0.001]

We first investigated whether the expression of Dectin-1 was altered in Ang II-related kidney injury. Kidney tissue lysates from mice infused with Ang II for 4 weeks were prepared, and as shown in Fig. 1A, B, the levels of Dectin-1 were significantly elevated following Ang II administration, which paralleled the mRNA expression of *Clec7a* in mouse kidneys (Fig. 1C). Moreover, immunofluorescence staining showed increased levels of Dectin-1 in mouse kidney tissues (Fig. 1D, 1E).

It has been reported that the expression of Dectin-1 is cell-specific; that is, Dectin-1 is mainly expressed by immune cells of myeloid origin [28]. To more accurately examine the function of Dectin-1, renal tissues were subjected to double immunofluorescence staining for Dectin-1 and markers of macrophages (CD68), glomerular cells (Wt1, Wilms Tumor), and renal tubular cells (AQP1, Aquaporin 1) to determine the distribution of Dectin-1 in the kidney. We found that CD68^+^ macrophages were the most abundant cell type expressing Dectin-1 in the kidney (Fig. 1F, G). In addition, we also measured the expression of Dectin-1 and Syk (the classic downstream kinase protein of Dectin-1) in bone marrow-derived monocytes (BMDMs), RAW 264.7 macrophage line, and renal SV40 cells. As shown in Fig. 1H, only BMDMs and RAW 264.7 macrophages expressed Dectin-1 and Syk.

Moreover, we investigated whether the level of Dectin-1 was altered in human hypertensive nephropathy. Similar to our findings, the public renal biopsy transcriptome database showed that *Clea7a* in HN was significantly upregulated compared to that in healthy donors (Fig. 1I). In addition, we further performed immunofluorescence staining in human kidney tissue samples from patients with HN or HL. The results validated that Dectin-1 protein was markedly increased in the kidneys of patients with HN (Fig. 1J, Supplementary Figure S1A).

Taken together, these findings suggest that Ang II upregulates Dectin-1 expression in the kidney, mostly in CD68-positive macrophages.

### Dectin-1 deficiency ameliorated renal fibrosis induced by Ang II

**Fig. 2:**
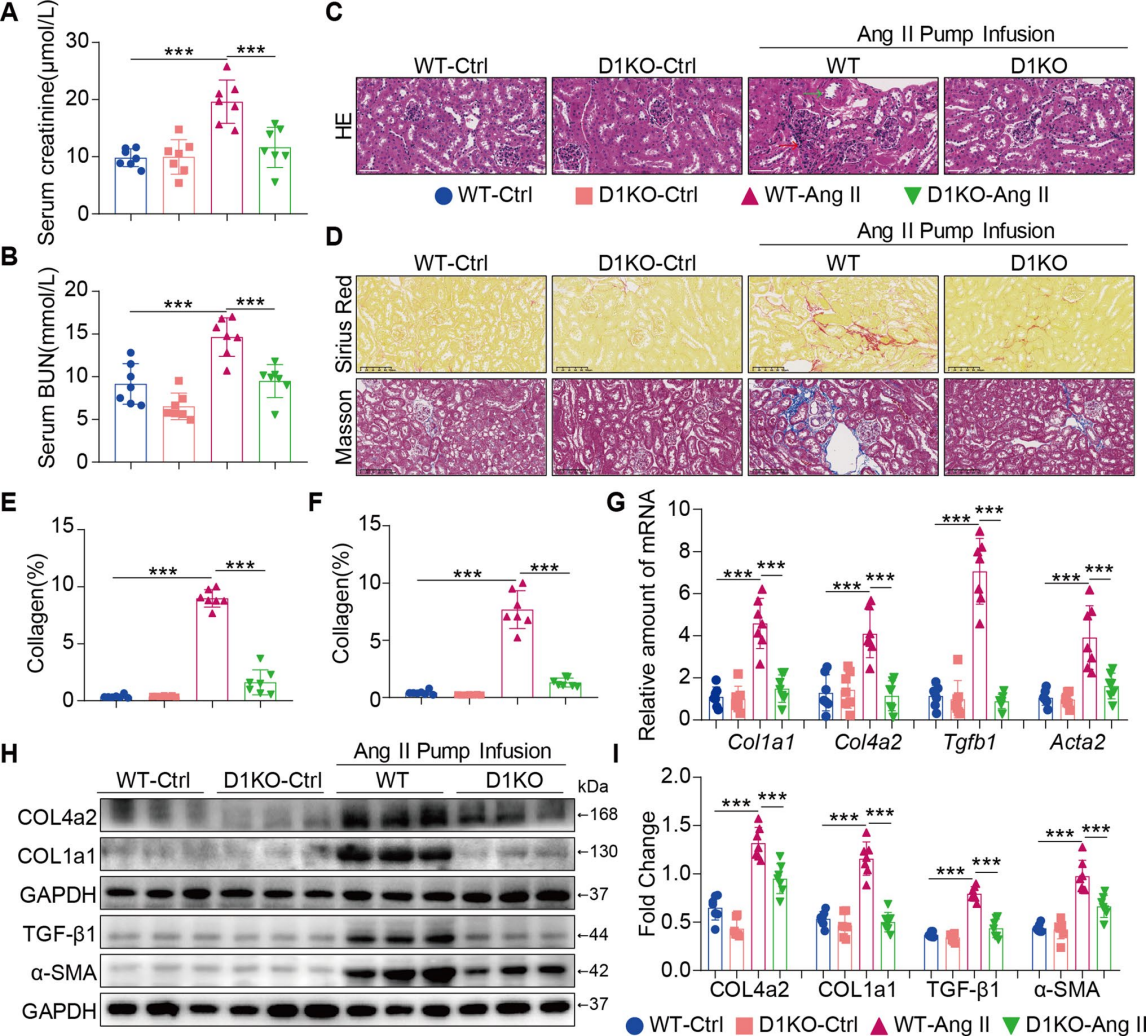
Dectin-1 deficiency ameliorated renal fibrosis induced by Ang II. **A** The serum creatinine levels in mice of each group. **B** The serum BUN levels in mice of each group. **C** Representative H&E staining of kidney tissues showing the effect of Dectin-1 deficiency on Ang II-induced kidney injury. **D** Fibrosis in kidney tissues of Ang II-challenged mice. SR panel shows representative micrographs of Sirius Red staining and Masson panel shows representative micrographs of Masson Trichrome staining. (n = 5–7; scale bar, 100 μm); **E**, **F** Quantification of interstitial fibrotic areas (%) from Sirius Red-stained kidney sections (E) and Masson’s Trichome staining (F); **G** Real-time qPCR showing mRNA levels of *Col1a1*, *Col4a2*, *Tgfb1 and Acta2* in the kidney tissues; **H** Representative western blot analysis of COL4A2, COL1A1, TGF-β1 and α-SMA in kidney tissues. GAPDH was used as loading control; **I** Densitometric quantification of immunoblots in Fig. 2H. Levels of COL4A2, COL1A1, TGF-β1 and α-SMA were normalized to GAPDH; [A-I, n = 5–7; 1-way ANOVA followed by Tukey post-hoc tests, (B-D, F-J: number of comparisons = 10). *P < 0.05, **P < 0.01, and ***P < 0.001]

To determine whether the increase in Dectin-1 expression could cause renal fibrosis, WT mice and D1KO mice were examined, and Ang II infusion (1000 ng/kg/min) for 4 weeks was used to establish renal injury. Firstly, these mice show barely detectable Dectin-1 protein (Supplementary Figure S2A) and mRNA (Supplementary Figure S2B) in kidney tissues. No differences of body weights were observed in the 4-week study (Supplementary Figure S2C) in each group. Nevertheless, 4 weeks of Ang II administration caused an elevated systolic blood pressure in WT and D1KO mice (Supplementary Figure S2D). Ang II infusion induced renal injury, as indicated by increased serum levels of creatinine and BUN, and Dectin-1 deficiency improved the impairment in kidney function (Fig. 2A, B). Moreover, Dectin-1 deficiency significantly reduced Ang II-induced renal injury (Fig. 2C, green arrow indicates thickened arteries; red arrow indicates infiltrated monocytes). We next examined the role of Dectin-1 in regulating renal fibrosis in WT and D1KO mice. Dectin-1 deficiency significantly alleviated Ang II-induced renal fibrosis (secretion of cell matrix and deposition of collagen) compared with that in WT mice (Fig. 2D–F). Accordingly, the mRNA and protein expression of Collagen 4A2, Collagen 1A1, α-SMA and TGF-β1 were reduced in D1KO mouse kidneys (Fig. 2G–I). These results demonstrated that Dectin-1 deficiency ameliorated renal fibrosis induced by Ang II.

### Dectin-1 deficiency alleviated macrophage migration, neutrophil infiltration and inflammation induced by Ang II infusion

**Fig. 3:**
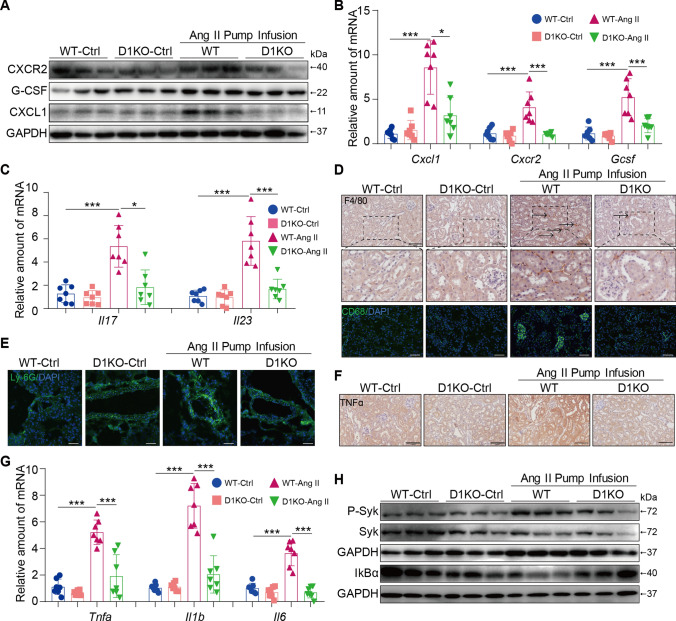
Dectin-1 deficiency alleviated macrophage migration, neutrophil infiltration and inflammation induced by Ang II infusion. **A** Representative western blot analysis of G-CSF, CXCL1, and CXCR2 in kidney tissues. GAPDH was used as loading control; **B** Real-time qPCR showing mRNA levels of *Cxcl1*, *Cxcr2* and *Gcsf* in the kidney tissues; **C** Real-time qPCR showing mRNA levels of *Il17* and *Il23* in the kidney tissues; **D** Representative immunohistochemical analyses of F4/80 (upper) and immunofluorescence staining of CD68 (down) in murine kidney of each group (n = 5; scale bar, 100 μm); **E** Representative immunofluorescence staining of Ly-6G in murine kidney of each group (n = 5; scale bar, 50 μm); **F** Representative immunohistochemical analyses of TNFα in murine kidney of each group (n = 5; scale bar, 100 μm); **G** Real-time qPCR showing mRNA levels of *Tnfa, Il1b* and *Il6* in the kidney tissues; **H** Representative western blot analysis of P-Syk, Syk and IκBα in kidney tissues. GAPDH was used as loading control; [A-F, n = 5–7; 1-way ANOVA followed by Tukey post-hoc tests, (A-F: number of comparisons = 10). *P < 0.05, **P < 0.01, and ***P < 0.001]

The CXCL1-CXCR2 axis is involved in macrophage migration induced by Ang II [29]. To investigate the potential mechanism by which Dectin-1 knockdown improves renal injury, we examined chemokine expression in each group. Our data showed that Ang II infusion increased the expression of CXCR2, G-CSF and CXCL1 in WT mouse kidneys, and these factors were markedly reduced in D1KO mouse kidneys (Fig. 3A, B, Supplementary Figure S2E). Similar results were found for the mRNA levels of *Il17* and *Il23* (Fig. 3C). Immunofluorescence and immunohistochemical staining further confirmed that Dectin-1 deletion in mice significantly abrogated Ang II-induced recruitment of proinflammatory cells (F4/80^+^ macrophages, CD68^+^ macrophages, Ly-6G^+^ neutrophils, CXCL1^+^CD68^+^ macrophages and G-CSF^+^CD68^+^ macrophages) (Fig. 3D–E, Supplementary Figure S2F-H, Supplementary Figure S3). In addition, TNF-α staining and quantitative real-time polymerase chain reaction (qPCR) analysis further confirmed that Dectin-1 deletion in mice markedly protected against the Ang II-induced expression of TNF-α, *Il1b* and *Il6* compared with those in WT controls (Fig. 3G and Supplementary Figure S2I).

We next examined which pathways were involved in renal injury and found that Ang II increased Syk phosphorylation and IκBα degradation, and these effects were markedly downregulated in D1KO mouse kidneys compared with WT controls (Fig. 3H and Supplementary Figure S2J). Thus, these data indicate that Dectin-1 mediates the accumulation of proinflammatory cells by activating the Syk-p65 pathway, which contributes to renal inflammation.

### Dectin-1/Syk promoted the expression and secretion of chemokines in Ang II-induced macrophages

Considering the critical role of Dectin-1 in mediating Ang II-induced renal injury, we next determined whether Dectin-1 influences macrophage activation in vitro. As we previously reported, Ang II acts as a new endogenous ligand of Dectin-1 and directly binds to Dectin-1 to induce Dectin-1 dimerization and Dectin-1/Syk pathway activation [30]. Here, we further validated the role of Dectin-1/Syk signaling in Ang II-induced macrophages activation.

**Fig. 4:**
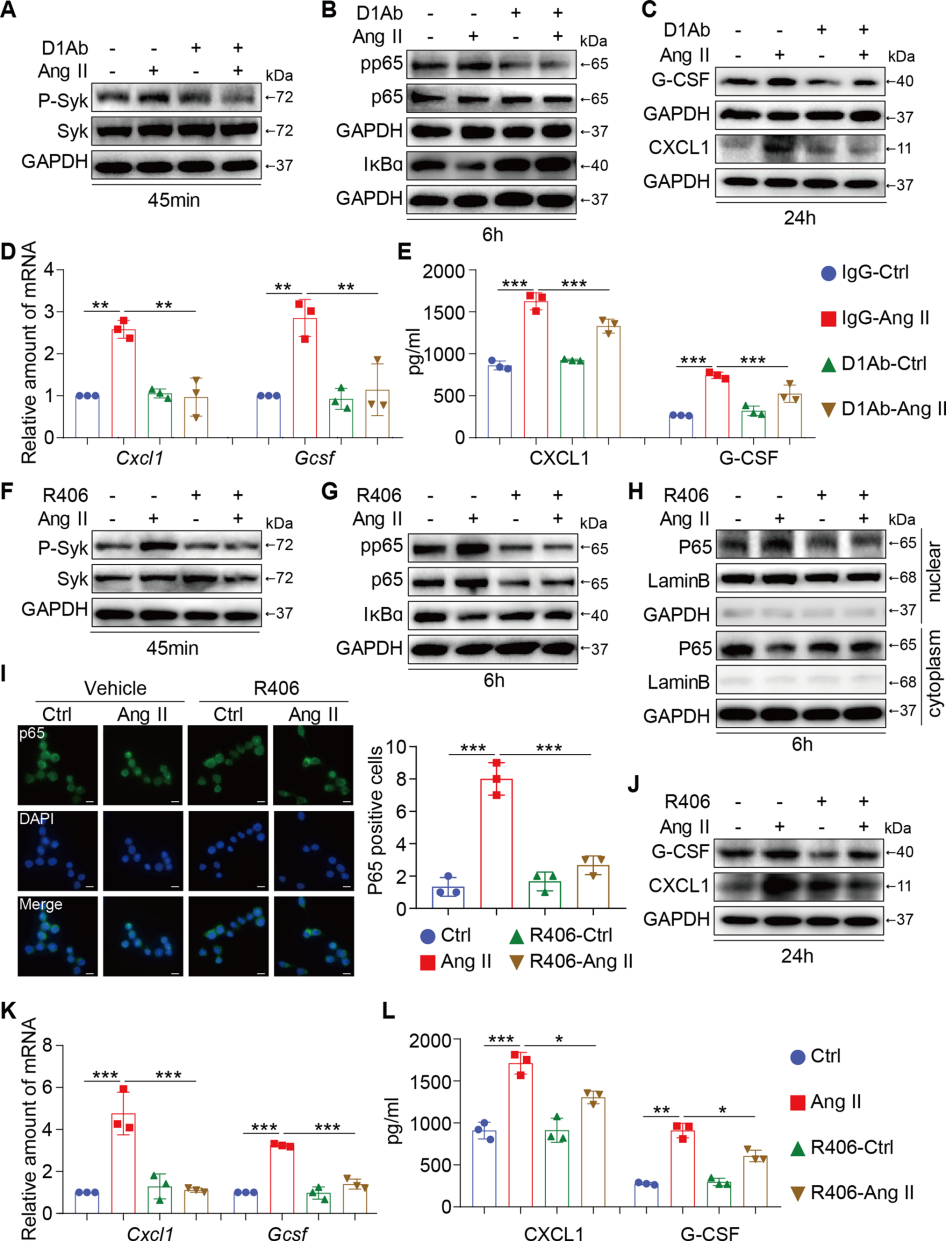
Dectin-1/Syk promoted the expression and secretion of chemokines in Ang II-induced macrophages. **A** RAW cells were treated with anti-Dectin-1 antibody (5 µg/mL) for 30 min, and then stimulated with Ang II (1 µM) for 45 min. Total proteins were extracted and subjected to analysis of P-Syk and Syk protein levels. GAPDH was used as loading control; **B** RAW cells were treated with anti-Dectin-1 antibody (5 µg/mL) for 30 min, and then stimulated with Ang II (1 µM) for 6 h. Total proteins were extracted and subjected to analysis of pp65, p65 and IκBα protein levels. GAPDH was used as loading control; **C** RAW cells were treated with anti-Dectin-1 antibody (5 µg/mL) for 30 min, and then stimulated with Ang II (1 µM) for 24 h. Total proteins were extracted and subjected to analysis of G-CSF, CXCL1 and TGF-β1 protein levels. GAPDH was used as loading control; **D** RAW cells were treated with anti-Dectin-1 antibody (5 µg/mL) for 30 min, and then stimulated with Ang II (1 µM) for 6 h. Real-time qPCR showing mRNA levels of *Cxcl1* (D, left) and *Cxcr2* (D, right) of each group; **E** RAW cells were treated with anti-Dectin-1 antibody (5 µg/mL) for 30 min, and then stimulated with Ang II (1 µM) for 24 h, CXCL1 (E, left) and G-CSF (E, right) protein secretion were determined in the supernatants level of each group by an ELISA method; **F** RAW cells were treated with Syk inhibitor (R406, 10 µmol/mL) for 1 h, and then stimulated with Ang II (1 µM) for 45 min. Total proteins were extracted and subjected to analysis of P-Syk and Syk protein levels. GAPDH was used as loading control; **G** RAW cells were treated with Syk inhibitor (R406, 10 µmol/mL) for 1 h, and then stimulated with Ang II (1 µM) for 6 h. Total proteins were extracted and subjected to analysis of pp65, p65 and IκBα protein levels. GAPDH was used as loading control; **H** Western blot detected P65, Lamin B and GAPDH protein levels in the nuclear extractions and cytoplasm.** I** Representative images of p65 nuclear translocation detected by immunofluorescence microscopy; The right panel was the quantification of p65 nuclear translocation. **J** RAW cells were treated with Syk inhibitor (R406, 10 µmol/mL) for 1 h, and then stimulated with Ang II (1 µM) for 24 h. Total proteins were extracted and subjected to analysis of G-CSF and CXCL1 protein levels. GAPDH was used as loading control; **K** RAW cells were treated with Syk inhibitor (R406, 10 µmol/mL) for 1 h, and then stimulated with Ang II (1 µM) for 6 h. Real-time qPCR showing mRNA levels of *Cxcl1* (K, left) and *Cxcr2* (G, right) of each group; **L** RAW cells were treated with Syk inhibitor (R406, 10 µmol/mL) for 1 h, and then stimulated with Ang II (1 µM) for 24 h. CXCL1 (L, left) and G-CSF (L, right) protein secretion were determined in the supernatants level of each group by an ELISA method; [A-K, I, n = 3; 1-way ANOVA followed by Tukey post-hoc tests (A-B: number of comparisons = 10; C-K number of comparisons = 6); *P < 0.05, **P < 0.01, and ***P < 0.001]

First, the Dectin-1 neutralizing antibody(D1Ab) and RAW264.7 cells were used. Blocking Dectin-1 with the D1Ab reduced the high levels of P-Syk in RAW 264.7 cells induced by Ang II (Fig. 4A and Supplementary Figure S4A). In addition, Dectin-1 blockade significantly inhibited Ang II-induced p65 pathway activation (inhibiting p65 phosphorylation and IκBα degradation) (Fig. 4B and Supplementary Figure S4B). Thus, the Sky-p65 pathway may be the key pathway involved in Dectin-1-mediated macrophage activation. In addition, prolonged exposure to Ang II significantly increased the expression and secretion of CXCL1 and G-CSF by RAW264.7 cells, and pretreatment with the D1Ab abrogated these increases compared with IgG pretreatment (Fig. 4C–E and Supplementary Figure S4C). Blocking Dectin-1 also inhibited macrophage inflammation in response to Ang II (Supplementary Figure S4D).

We identified an essential role for Syk as a key protein in the modulation of Ang II-induced Dectin-1 activation in macrophages. Next, the Syk-specific inhibitor R406 was used to examine the biological functions of Syk further. Pretreatment with R406 inhibited Ang II-induced Syk phosphorylation in vitro (Fig. 4F and Supplementary Figure S4E). Moreover, in parallel with the effects of the D1Ab, the Ang II-induce activation of the p65 pathway (inhibiting p65 phosphorylation, IκBα degradation, and nuclear transfer of p65) was significantly reduced by the administration of R406 (Fig. 4G–I and Supplementary Figure S4F-G), indicating that p65 is downstream of Dectin-1-Syk. Initially, RAW 264.7 cells were stimulated with Ang II, and the expression and secretion of CXCL1 and G-CSF were significantly enhanced. Similar to the effects of the D1Ab, the high expression of these cytokines was greatly decreased when Syk was inhibited (Fig. 4J–L and Supplementary Figure S4H). Moreover, inhibiting Syk also alleviated Ang II-induced expression of inflammatory factors in macrophages (Supplementary Figure S4I). These results suggest that Dectin-1/Syk promoted the expression and secretion of chemokines in Ang II-induced macrophages and that these secreted cytokines may be the key mediators of renal injury induced by Ang II.

### Inhibiting Dectin-1/Syk decreased the TGF-β1-signaling pathway

**Fig. 5:**
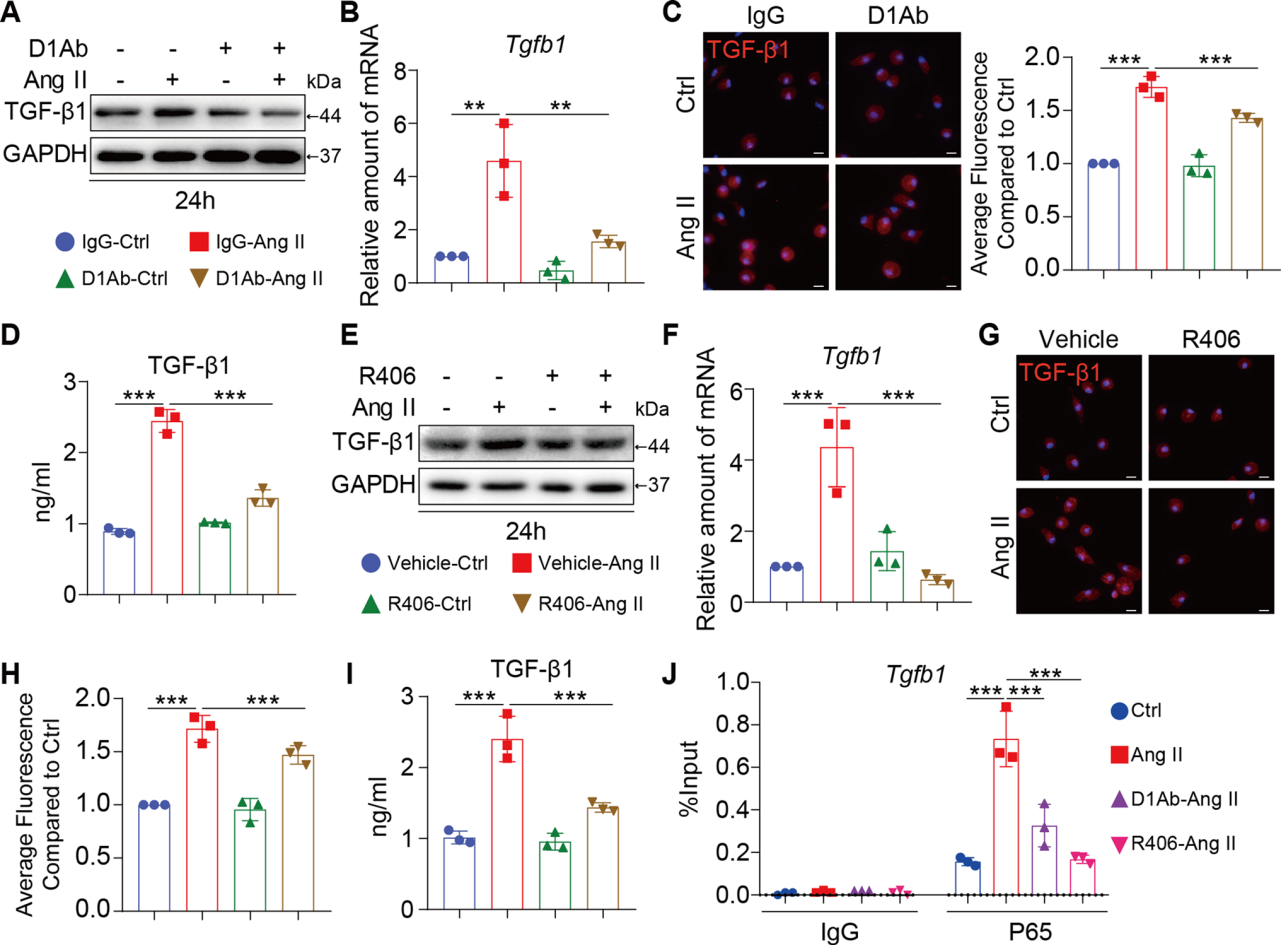
Inhibiting Dectin-1/Syk decreased the TGF-β1-signaling pathway. **A** RAW cells were treated with anti-Dectin-1 antibody (5 µg/mL) for 30 min, and then stimulated with Ang II (1 µM) for 24 h. Total proteins were extracted and subjected to analysis of TGF-β1 protein levels. GAPDH was used as loading control; **B** RAW cells were treated with anti-Dectin-1 antibody (5 µg/mL) for 30 min, and then stimulated with Ang II (1 µM) for 6 h. Real-time qPCR showing mRNA levels of *tgfb1* of each group; **C** RAW cells were treated with anti-Dectin-1 antibody (5 µg/mL) for 30 min, and then stimulated with Ang II (1 µM) for 24 h. Representative images of TGF-β1 protein expression detected by immune fluorescence microscopy. The right panel shown the quantification of the average fluorescence as detected by immunofluorescence staining compared to Ctrl group. **D** RAW cells were treated with anti-Dectin-1 antibody (5 µg/mL) for 30 min, and then stimulated with Ang II (1 µM) for 24 h. TGF-β1 protein secretion were determined in the supernatants level of each group by an ELISA method; **E** RAW cells were treated with Syk inhibitor (R406, 10 µmol/mL) for 1 h, and then stimulated with Ang II (1 µM) for 24 h. Total proteins were extracted and subjected to analysis of TGF-β1 protein levels. GAPDH was used as loading control; **F** RAW cells were treated with Syk inhibitor (R406, 10 µmol/mL) for 1 h, and then stimulated with Ang II (1 µM) for 6 h. Real-time qPCR showing mRNA levels of *tgfb1* of each group; **G** RAW cells were treated with Syk inhibitor (R406, 10 µmol/mL) for 1 h, and then stimulated with Ang II (1 µM) for 24 h. Representative images of TGF-β1 protein expression detected by immune fluorescence microscopy; **H** The quantification of the average fluorescence as detected by immunofluorescence staining compared to Ctrl group in Fig. 5G. **I** RAW cells were treated with Syk inhibitor (R406, 10 µmol/mL) for 1 h, and then stimulated with Ang II (1 µM) for 24 h. TGF-β1 protein secretion were determined in the supernatants level of each group by an ELISA method; **J** ChIP-qPCR analysis of blocking Dectin-1(D1Ab) and Syk antagonist (R406) inhibited the p65 binding to Tgfb1 promoter induced by Ang II in RAW cells, respectively. [A-J, n = 3; 1-way ANOVA followed by Tukey post-hoc tests (A-J: number of comparisons = 6; *P < 0.05, **P < 0.01, and ***P < 0.001]

Considering the role of Dectin-1 deficiency in attenuating renal fibrosis in vivo, we then examined the underlying mechanism of this renal protection. TGF-β1, which is a critical cytokine, significantly induces cellular fibrosis [31, 32]. Compared with those in Ang II-stimulated RAW 264.7 cells, the protein and mRNA expression of TGF-β1 were markedly lower in cells that were pretreated with the D1Ab (Fig. 5A, B and Supplementary Figure S5A). Using immunofluorescent staining, we found that Ang II stimulation significantly increased the fluorescence intensity of TGF-β1 compared with that in the IgG-Ctrl group, and this effect was reversed by Dectin-1 blockade (Fig. 5C and Supplementary Figure S5A). Moreover, the level of TGF-β1 in the supernatant was reduced by D1Ab treatment (Fig. 5D). Similarly, inhibiting SYK decreased the expression and secretion of TGF-β1 (Fig. 5E–I and Supplementary Figure S5B). Based on the marked attenuation of P65 pathway activation in the presence of D1Ab and R406, a ChIP–qPCR assay was performed. Ang II significantly increased the binding of P65 to the TGF-β1 promoter, indicating that P65 activated the transcription of TGF-β1. In addition, the binding of P65 to the TGF-β1 promoter was significantly reduced by blocking Dectin-1 or inhibiting Syk, which may correlate with the reduction in P65 entry into the nucleus (Fig. 5J). Thus, Dectin-1 could promote TGF-β1 expression in Ang II-induced macrophages via the Syk/p65 pathway and p65 binding to the promoter.

### Supernatant from Dectin-1/Syk-inhibited macrophages alleviated renal fibrosis

**Fig. 6:**
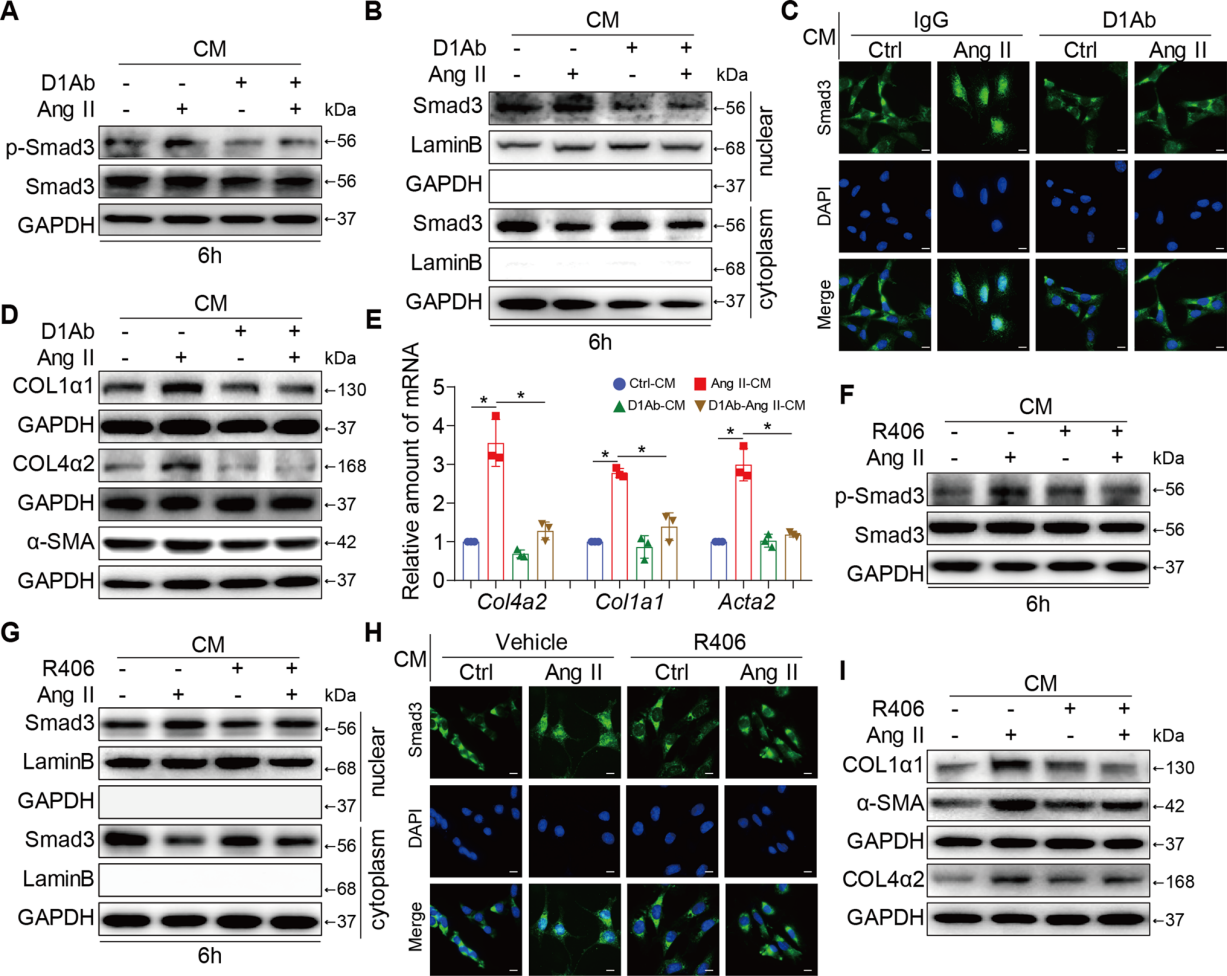
Supernatant containing Dectin-1/Syk inhibited macrophages alleviated renal fibrosis in vitro. SV40 cells were incubated with the supernatants from the RAW cells fellow the approach described in Figs. 4, 5. **A** SV40 cells were treated with the supernatants from the RAW cells fellow the approach described in Fig. 5A for 6 h. Total proteins were extracted and subjected to analysis of p-Smad3 and Smad3 protein levels. GAPDH was used as loading control; **B** Western blot detected Smad3, Lamin B and GAPDH protein levels in the nuclear extractions and cytoplasm. **C** Representative images of Smad3 nuclear translocation detected by immune fluorescence microscopy; **D** Representative western blot analysis of COL1α1, COL4α2 and α-SMA of each group in SV40 cells. GAPDH was used as loading control; **E** Real-time qPCR showing mRNA levels of *Col4a2, Col1a1* and *Acta2* of each group in SV40 cells.; **F** SV40 cells were treated with the supernatants from the RAW cells fellow the approach described in Fig. 5E for 6 h. Total proteins were extracted and subjected to analysis of p-Smad3 and Smad3 protein levels. GAPDH was used as loading control; **G** Western blot detected Smad3, Lamin B and GAPDH protein levels in the nuclear extractions and cytoplasm. **H** Representative images of Smad3 nuclear translocation detected by immune fluorescence microscopy; **I** Representative western blot analysis of COL1α1, COL4α2 and α-SMA of each group in SV40 cells. GAPDH was used as loading control; [A-I, n = 3; 1-way ANOVA followed by Tukey post-hoc tests (A-J: number of comparisons = 6; *P < 0.05, **P < 0.01, and ***P < 0.001]

To further examine whether the pathogenic role of macrophage-derived Dectin-1 in Ang II-related renal fibrosis was achieved by increasing TGF-β1 expression, SV40 cells, a glomerulus myofibroblast-like cell line [27], were cultured with the supernatant from Dectin-1/Syk-inhibited macrophages. We found that the supernatant of the Ang II-induced cells led to a significant increase in Smad3 phosphorylation and nuclear translocation, indicating activation of the TGF-β1/Smad3 pathway, while these effects were significantly reversed by Dectin-1 blockade in macrophages (Fig. 6A–C and Supplementary Figure S6A-C). In addition, this profibrotic effect was also markedly prevented in supernatant of macrophages containing the D1Ab (Fig. 6D, E and Supplementary Figure S6D). The supernatant from macrophages treated with R406 to inhibit Syk also alleviated renal TGF-β1/Smad3 pathway activation and blocked the expression of fibrotic genes and proteins (COL1α1, COL4α2 and α-SMA) induced by the Ang II-conditioned supernatant (Fig. 6F-I and Supplementary Figure S6E-I). This indirect culture model revealed that the supernatant of macrophages treated with Dectin-1 or Syk inhibitors could alleviate the profibrotic effects on SV40 cells, suggesting that macrophage Dectin-1/Syk may indirectly participate in macrophage-to-glomerulus fibroblast crosstalk mainly through regulating TGF-β1 production.

## Discussion

In this study, we demonstrated for the first time that macrophage-derived Dectin-1 mediates renal neutrophil migration and TGF-β1 secretion, which are critical pathogenic effects in Ang II-induced renal dysfunction and fibrosis. The levels of Dectin-1 in the kidney were significantly upregulated by Ang II infusion. Dectin-1 deficiency protected kidney function, inhibited neutrophil migration, and attenuated renal fibrosis and inflammation in Ang II-challenged mice. Consistently, in RAW264.7 cells, therapeutic blockade of Dectin-1 or Syk inhibition markedly attenuated Ang II-induced expression and secretion of chemokines and TGF-β1, which in turn protected SV40 cells from TGF-β1/Smad3 activation. Thus, our evidence suggests a powerful pathogenic role of macrophage-derived Dectin-1 in the pathogenesis of hypertensive renal diseases.

Previous studies have reported that Dectin-1, which was originally described as a β-glucan receptor, is mostly expressed in myeloid cells [33]. In our study, using immunofluorescence double-staining, we validated that in the kidney, Dectin-1 was expressed on CD68^+^ macrophages. This cell-specific expression was further characterized in vitro, using BMDMs, RAW264.7, and SV40 cells. It is guessed that the phenotypes of whole-body Dectin-1 knockout mice should be similar with that of macrophage-specific Dectin-1 knockout mice. In fact, all previously published papers on Dectin-1 used the whole-body Dectin-1 KO mice. Adverse increases in chemokines have been reported to be characteristic of hypertensive kidney disease and affect kidney injury [34]. IL-18 is produced by monocytes and macrophages and increases renal mRNA expression of the proinflammatory cytokines TNF-α, CXCL2, CCL2, as well as tubular injury, after bilateral I/R injury in mice [35]. CXCR1 produced by dendritic cells exerts protective effects by modulating the invasion of inflammatory cells in hypertensive renal injury [36]. CXCR6 produced by T cells, monocytes, and myeloid fibroblasts promotes the development of Ang II-induced renal injury and fibrosis through the regulation of macrophage and T-cell infiltration and bone marrow-derived fibroblast accumulation [37]. In our study, Ang II infusion markedly increased the levels of chemokines (CXCL1 and GCSF) and CXCR2 (the receptor of CXCL1) in the kidney, and Dectin-1 deficiency attenuated Ang II-induced renal dysfunction and renal fibrosis. In addition, the expression of chemokines (CXCL1 and GCSF) was reduced in D1KO mice compared with Ang II-infused mice, which in turn alleviated Ly-6G^+^ neutrophil infiltration and renal inflammation.

It is well established that Syk, which is the fundamental kinase in the activation pathway, is triggered by Dectin-1 to activate the downstream pathway [38]. Several downstream effectors are activated. One of the most pleiotropic signaling pathways is the phosphatidylinositol 3-kinase (PI3K)/AKT pathway [39, 40]. In addition, phospholipase C-γ2 (PLC-γ2) and Ca2^+^-dependent activation are followed by Syk phosphorylation [41]. In addition, Olaf Gross et al. showed that Syk activation is required for the formation of a signaling complex composed of caspase recruitment domain 9 (CARD9), a scaffold molecule that binds to B-cell lymphoma 10 (BCL10) and forms a trimolecular complex with BCL10 and mucosa-associated lymphoid tissue 1 (MALT1) [42]. This complex promoted nuclear factor kappa-B (NF-κB) activation, eventually leading to the production of inflammatory cytokines such as pro-IL-1β, IL-6 and TNF-α [33, 42]. Here, we showed that Dectin-1 deficiency reduced Syk phosphorylation and IκBα degradation in Ang II-infused kidneys, and blocking Dectin-1 or inhibiting Syk attenuated p65 phosphorylation, IκBα degradation, and nuclear transfer of p65 in RAW cells exposed to Ang II. These results indicated that Dectin-1/Syk/P65 may be the key pathway involved in Ang II-related macrophage activation.

In the kidney, almost all forms of CKD inevitably result in progressive interstitial fibrosis and reach the same endpoint: kidney failure [43]. TGF-β1 initiates collagen deposition, which drives interstitial fibrosis [44]. Livingston MJ and colleagues showed that fibroblast growth factor 2 (FGF2) production in tubular cells mediated renal fibrosis through the secretion of TGF-β1 to activate fibroblasts for renal fibrosis following acute kidney injury (AKI) [45]. Ang II induced myofibroblasts to upregulate intracellular expression and secretion of TGF-β1 [46]. However, whether Dectin-1/Syk promotes Ang II-related renal fibrosis via TGF-β1 is still unknown. In the current study, D1KO mice were generated to assess the potential roles of Dectin-1 in renal injury induced by Ang II, especially renal fibrosis. We found that Dectin-1 deletion suppressed TGF-β1 production in the kidney and alleviated renal fibrosis. Mechanistically, blocking Dectin-1 or inhibiting Syk reduced the increases in TGF-β1 expression and secretion in vivo, as evidenced by the reduced binding of P65 to the TGF-β1 promoter. In addition, the conditioned medium experiments revealed that Dectin-1/Syk may participate in macrophage-to-glomerulus myofibroblast crosstalk mainly through the TGF-β1/Smad3 pathway.

### Perspectives

The key findings of our study demonstrated for the first time that macrophage-derived Dectin-1 activated neutrophil migration into the kidney and upregulated TGF-β1 secretion, leading to renal dysfunction and fibrosis in response to Ang II infusion. Prophylactic blockade of Dectin-1/Syk signaling prevented adverse renal inflammation, fibrosis and dysfunction, thus representing an attractive new strategy for treating hypertensive renal injury. An unanswered but important question is how Ang II activates Dectin-1 in macrophages, which needs future studies to dissect out the precise molecular mechanism between Ang II and Dectin-1 interaction.

### Supplementary Information

Below is the link to the electronic supplementary material.

Supplementary file1 (DOCX 4216 KB)

## Data Availability

All other data and materials are included within the article or Supplementary Information or available from the authors on request.

## Abbreviations

Ang II: Angiotensin II
ACE: Angiotensin-converting enzyme
ARBs: Angiotensin II receptor blockers
aTRH: Apparent treatment-resistant hypertension
AQP1: Aquaporin 1
BMDM: Bone marrow-derived monocytes
CLRs: C‐type lectin receptors
ChIP: Chromatin immunoprecipitation
CKD: Chronic kidney disease
COL1A1: Collagen type I alpha 1 chain
COL4A2: Collagen type IV alpha 2 chain
CXCL1: C-X-C motif ligand 1
CXCR2: CXC receptor 2
D1KO mice: Dectin-1^−/−^ mice
D1Ab: Dectin-1 neutralizing antibody
EAE: Experimental autoimmune encephalomyelitis
HL: Healthy living donor
HN: Hypertensive nephropathy
NF-κB: Nuclear factor kappa-B
ROI: Region of interest
TGF-β1: Transforming growth factor beta 1
Wt1: Wilms tumor
