# Supplementary material for: Macrophage Dectin-1 mediates Ang II renal injury through neutrophil migration and TGF-β1 secretion
Source: Cell Mol Life Sci. 2023 Jun 20;80(7):184. doi: 10.1007/s00018-023-04826-4 (PMC10282045; doi:10.1007/s00018-023-04826-4)
Supplement: Supplementary file 1 — Supplementary file1 (DOCX 4216 KB) [file 18_2023_4826_MOESM1_ESM.docx]

*Supplement Materials*

**Macrophage Dectin-1 mediates** **angiotensin II-induced renal injury by activating neutrophil migration and TGF-β1 secretion**

**Running headline:** Dectin-1 mediates Ang II-induced renal injury

**Supplemental File:** 1 Table, and 6 Figures

**Supplementary Tables and Figures**

**Supplementary Table S1. Primer sequences for qPCR.**

| **Gene** | **Species** | **Sequence** |
| --- | --- | --- |
| *Col4a2* | Mouse | CTGGCACAAAAGGGACGAG  ACGTGGCCGAGAATTTCACC |
| *Col1a1* | Mouse | GCTCCTCTTAGGGGCCACT  CCACGTCTCACCATTGGGG |
| *Tgfb1* | Mouse | CTCCCGTGGCTTCTAGTGC  GCCTTAGTTTGGACAGGATCTG |
| *Acta2* | Mouse | GTCCCAGACATCAGGGAGTAA  TCGGATACTTCAGCGTCAGGA |
| *Clec7a* (Dectin-1) | Mouse | GACTTCAGCACTCAAGACATCC  TTGTGTCGCCAAAATGCTAGG |
| *Il17a* | Mouse | TTTAACTCCCTTGGCGCAAAA  CTTTCCCTCCGCATTGACAC |
| *Il23* | Mouse | ATGCTGGATTGCAGAGCAGTA  ACGGGGCACATTATTTTTAGTCT |
| *Cxcl1* | Mouse | CTGGGATTCACCTCAAGAACATC  CAGGGTCAAGGCAAGCCTC |
| *Cxcr2* | Mouse | ATGCCCTCTATTCTGCCAGAT  GTGCTCCGGTTGTATAAGATGAC |
| *Csf3* | Mouse | ATGGCTCAACTTTCTGCCCAG CTGACAGTGACCAGGGGAAC |
| *Tnfa* | Mouse | TGATCCGCGACGTGGAA ACCGCCTGGAGTTCTGGAA |
| *Il1b* | Mouse | ACTCCTTAGTCCTCGGCCA  CCATCAGAGGCAAGGAGGAA |
| *Il16* | Mouse | GAGGATACCACTCCCAACAGACC  AAGTGCATCATCGTTGTTCATACA |
| *Actb* | Mouse | GGCTGTATTCCCCTCCATCG  CCAGTTGGTAACAATGCCATGT |
| *Tgfb1* Promotor  (25385334-25386054) | Mouse | GTTTCAATGCTGGGAACCCG  GGGCAGACTTTGCGGATGA |

**
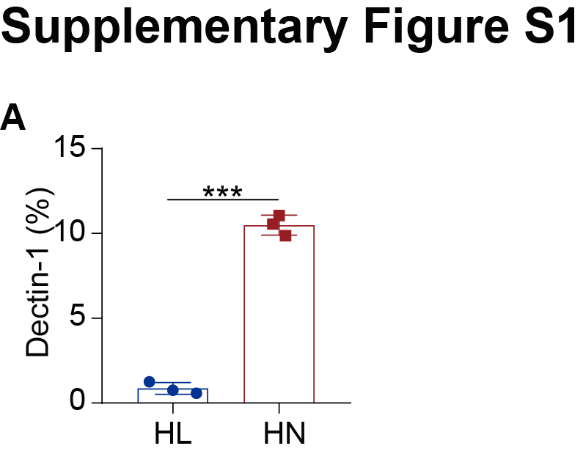
**

**Supplement Figure S1:** **Dectin-1 was markedly increased in the hypertensive nephropathy. Related to Figure 1**

(A): Quantification of Dectin-1 positive areas (%) from immunofluorescence staining of Dectin-1 in Figure 1J [n =3]

**
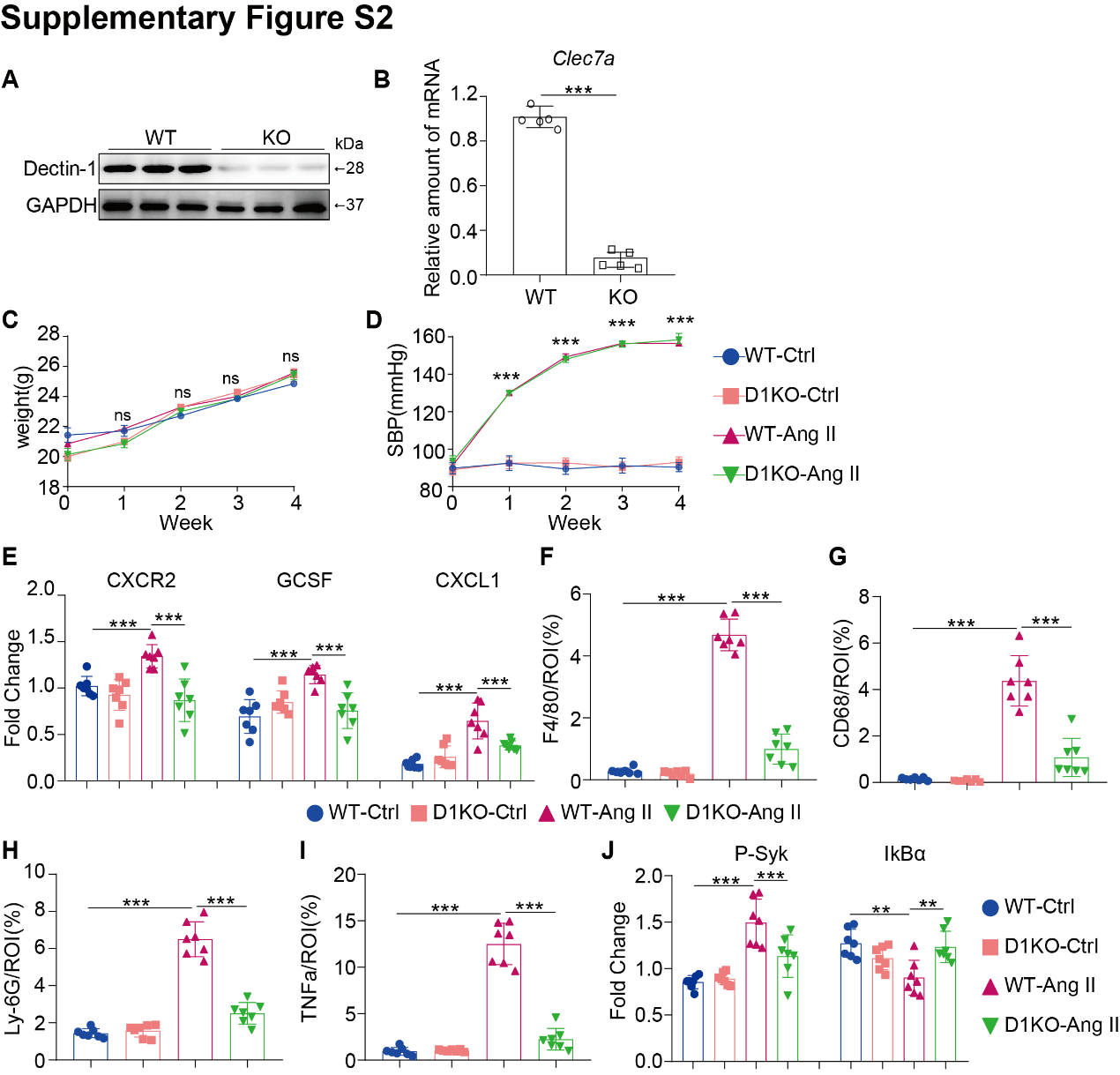
Supplement Figure S2:** **Dectin-1 deficiency alleviated the macrophage migration, neutrophil infiltration and inflammation induced by Ang II infusion. Related to Figure 2 and Figure 3.**

(A) Representative western blot analysis of Dectin-1 protein in kidney tissue from wildtype (WT) C57BL/6 and Dectin-1 knockout (KO) mice. GAPDH was used as loading control [n = 5].

(B) mRNA levels of *Clec7a* (Dectin-1) in the kidney tissue from WT mice and Dectin-1 KO mice [n = 5].

Quantification of Dectin-1 positive areas (%) from immunofluorescence staining of Dectin-1 in Figure 1J [n =3]

(C) Wildtype (WT) and Dectin-1 knockout (D1KO) mice were challenged with Ang II for 4 weeks using osmotic pumps. Body weights were measured over the experimental period [n = 7].

(D) Systolic blood pressure measurements were made weekly by non-invasive tail-cuff Pressure Analysis System in mice [n = 7].

(E) Densitometric quantification of immunoblots in Figure 3A. Levels of G-CSF, CXCL1, and CXCR2 were normalized to GAPDH; [n=7, one-way ANOVA followed by Tukey post-hoc tests, number of comparisons =6, *P<0.05, **P<0.01, and ***P<0.001].

(F) Quantification of F4/80 (F) positive areas (%) from immunohistochemical analyses of Figure 3D. [n=5-7, one-way ANOVA followed by Tukey post-hoc tests, number of comparisons =6, *P<0.05, **P<0.01, and ***P<0.001].

(G) Quantification of CD68 positive areas (%) from immunofluorescence staining of Figure 3D. [n=5-7, one-way ANOVA followed by Tukey post-hoc tests, number of comparisons =6, *P<0.05, **P<0.01, and ***P<0.001].

(H) Quantification of Ly-6G positive areas (%) from immunofluorescence staining of Figure 3E. [n=5-7, one-way ANOVA followed by Tukey post-hoc tests, number of comparisons =6, *P<0.05, **P<0.01, and ***P<0.001].

(I) Quantification of TNF-αpositive areas (%) from immunohistochemical analyses Figure 3F. [n=5-7, one-way ANOVA followed by Tukey post-hoc tests, number of comparisons =6, *P<0.05, **P<0.01, and ***P<0.001].

(J) Densitometric quantification of immunoblots in Figure 3H showing P-Syk: Syk, IκBα: GAPDH, [n=5-7, one-way ANOVA followed by Tukey post-hoc tests, number of comparisons =6, *P<0.05, **P<0.01, and ***P<0.001]

**
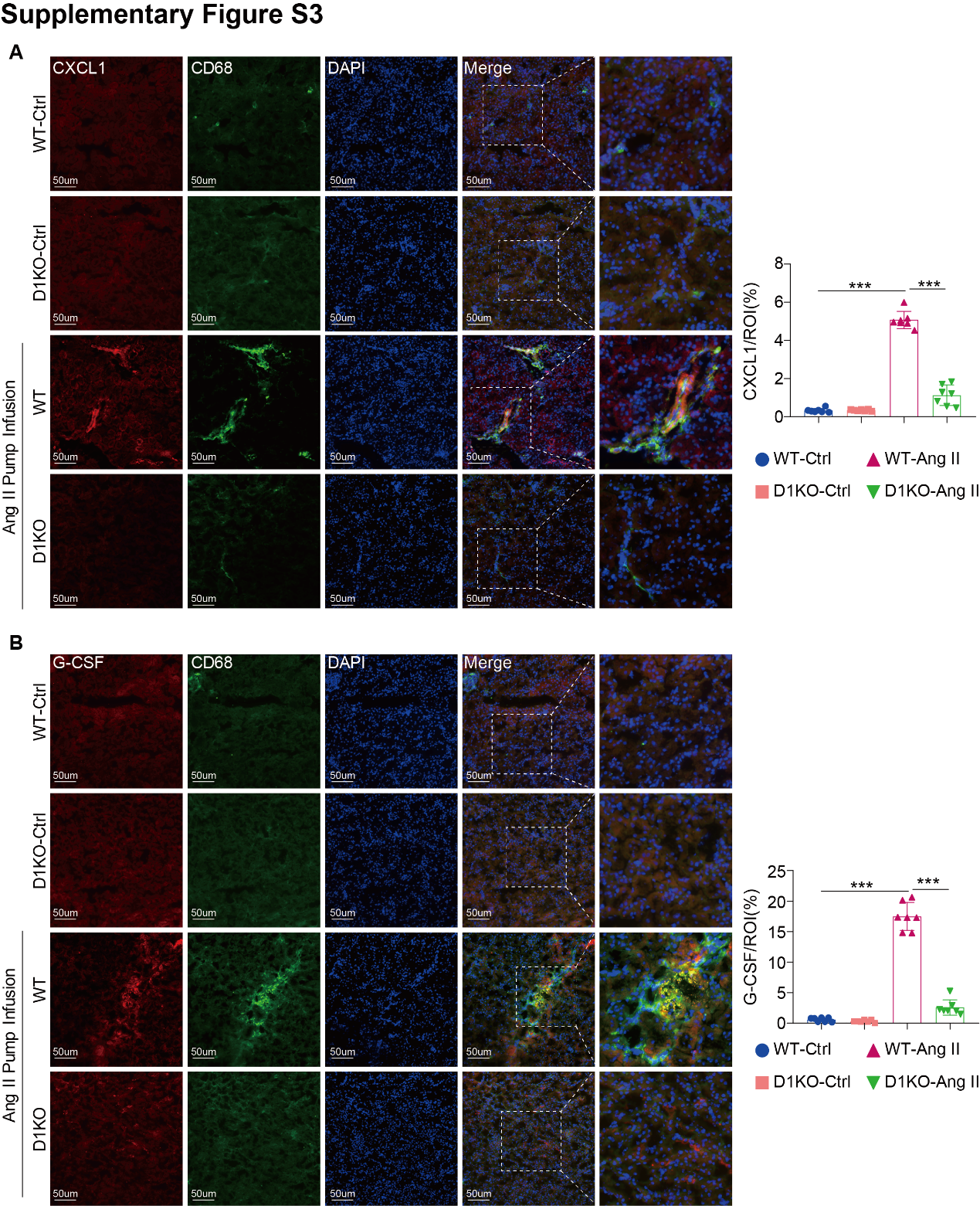
Supplement Figure S3:** **Dectin-1 induced macrophages infiltration after Ang II infusion. Related to Figure 3**

(A) Representative dual-immunofluorescence staining of CXCL1 and CD68 in murine kidney from each group (n=5; scale bar, 50μm); The right panel shows the quantification of CXCL1 positive areas (%) of ROI from dual-immunofluorescence staining of CXCL1 and CD68 of Online Figure IV A. [n=5-7, one-way ANOVA followed by Tukey post-hoc tests, number of comparisons =6, *P<0.05, **P<0.01, and ***P<0.001]

(B) Representative dual-immunofluorescence staining of G-CSF and CD68 in murine kidney from each group (n=5; scale bar, 50μm); The right panel shows the Quantification of G-CSF positive areas (%) of ROI from dual-immunofluorescence staining of G-CSF and CD68 of Online Figure IV B. [n=5-7, one-way ANOVA followed by Tukey post-hoc tests, number of comparisons =6, *P<0.05, **P<0.01, and ***P<0.001].

**
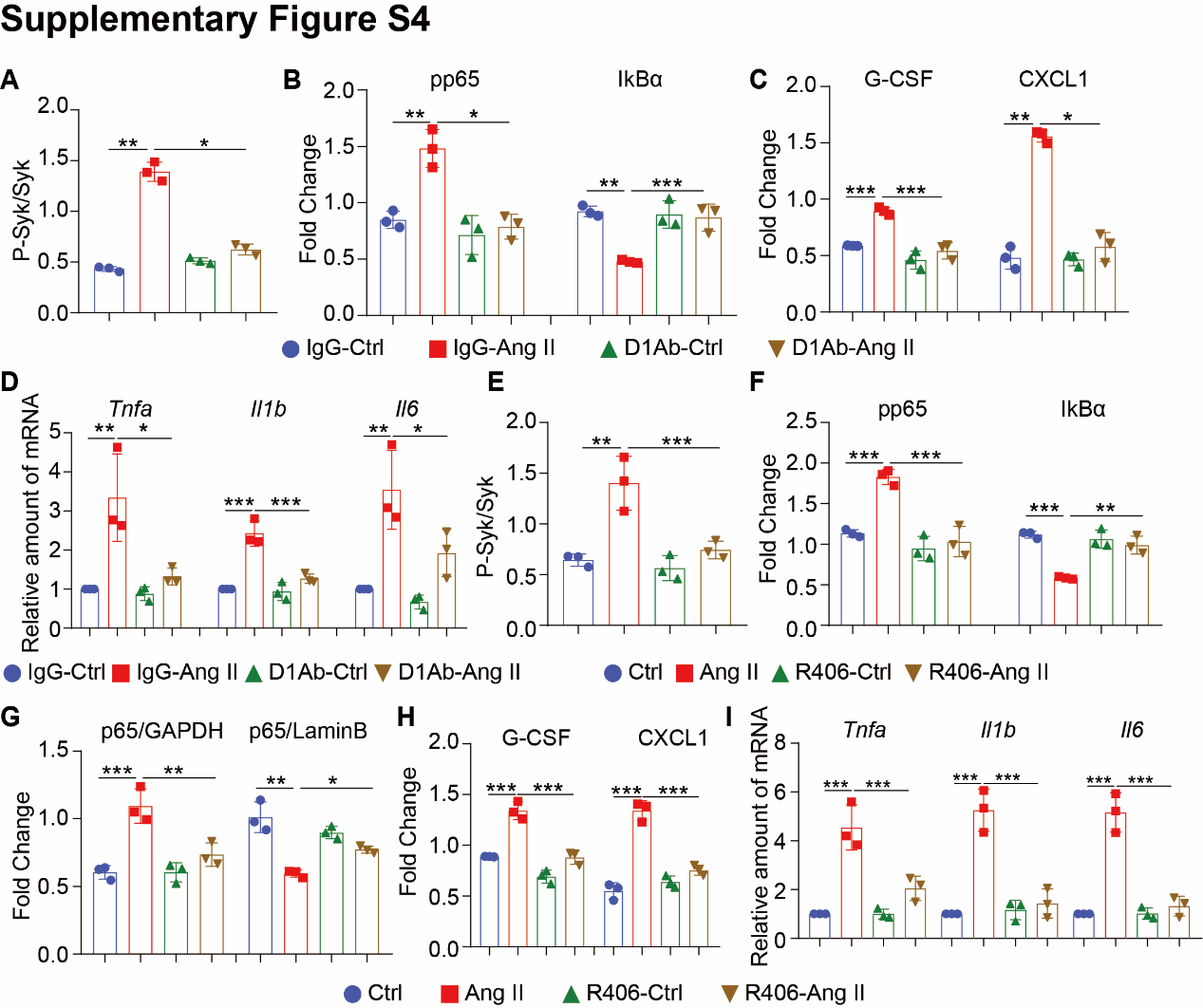
Supplement Figure S4: Dectin-1/Syk promoted the expression and secretion of chemokine in macrophage induced by Ang II. Related to Figure 4.**

(A) Densitometric quantification of immunoblots in in Figure 4A showing P-Syk: Syk. [n=3, one-way ANOVA followed by Tukey post-hoc tests, number of comparisons=6, *P<0.05, **P<0.01, and ***P<0.001].

(B) Densitometric quantification of immunoblots in in Figure 4B showing PP65: P65 and IκBα: GAPDH. [n=3, one-way ANOVA followed by Tukey post-hoc tests, number of comparisons=6, *P<0.05, **P<0.01, and ***P<0.001].

(C) Densitometric quantification of immunoblots in Figure 4C showing G-CSF: GAPDH and CXCL1: GAPDH [n=3, one-way ANOVA followed by Tukey post-hoc tests, number of comparisons=6, *P<0.05, **P<0.01, and ***P<0.001].

(D Real-time qPCR showing mRNA levels of *Tnfa*, *Il1b* and *Il6* of each group. [n=3, one-way ANOVA followed by Tukey post-hoc tests, number of comparisons=6, *P<0.05, **P<0.01, and ***P<0.001].

(E) Densitometric quantification of immunoblots in in Figure 4F showing P-Syk: Syk. [n=3, one-way ANOVA followed by Tukey post-hoc tests, number of comparisons=6, *P<0.05, **P<0.01, and ***P<0.001].

(F) Densitometric quantification of immunoblots in in Figure 4G showing PP65: P65 and IκBα: GAPDH. [n=3, one-way ANOVA followed by Tukey post-hoc tests, number of comparisons=6, *P<0.05, **P<0.01, and ***P<0.001].

(G) Densitometric quantification of immunoblots in Figure 5H showing P65: LaminB and P65: GAPDH [n=3, one-way ANOVA followed by Tukey post-hoc tests, number of comparisons=6, *P<0.05, **P<0.01, and ***P<0.001].

(H) Densitometric quantification of immunoblots in Figure 4G showing G-CSF: GAPDH and CXCL1: GAPDH [n=3, one-way ANOVA followed by Tukey post-hoc tests, number of comparisons=6, *P<0.05, **P<0.01, and ***P<0.001].

(I) Real-time qPCR showing mRNA levels of *Tnfa*, *Il1b* and *Il6* of each group. [n=3, one-way ANOVA followed by Tukey post-hoc tests, number of comparisons=6, *P<0.05, **P<0.01, and ***P<0.001].

**
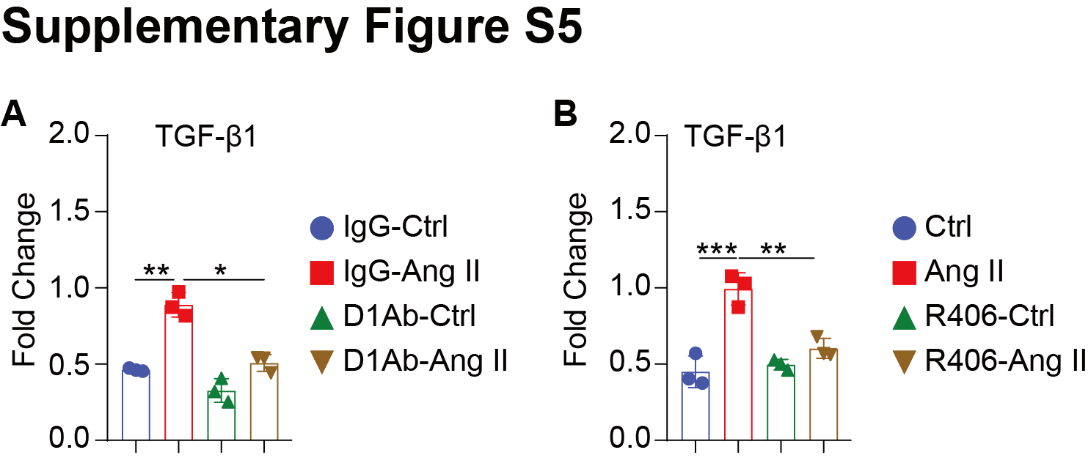
**

**Supplement Figure S5:** **Inhibiting Dectin-1/Syk decreased TGF-β1-Related Signaling Pathway. Related to Figure 5.**

(A) Densitometric quantification of immunoblots in Figure 5A showing TGF-β1: GAPDH [n=3, one-way ANOVA followed by Tukey post-hoc tests, number of comparisons=6, *P<0.05, **P<0.01, and ***P<0.001].

(C) Densitometric quantification of immunoblots in Figure 5E showing TGF-β1: GAPDH [n=3, one-way ANOVA followed by Tukey post-hoc tests, number of comparisons=6, *P<0.05, **P<0.01, and ***P<0.001].


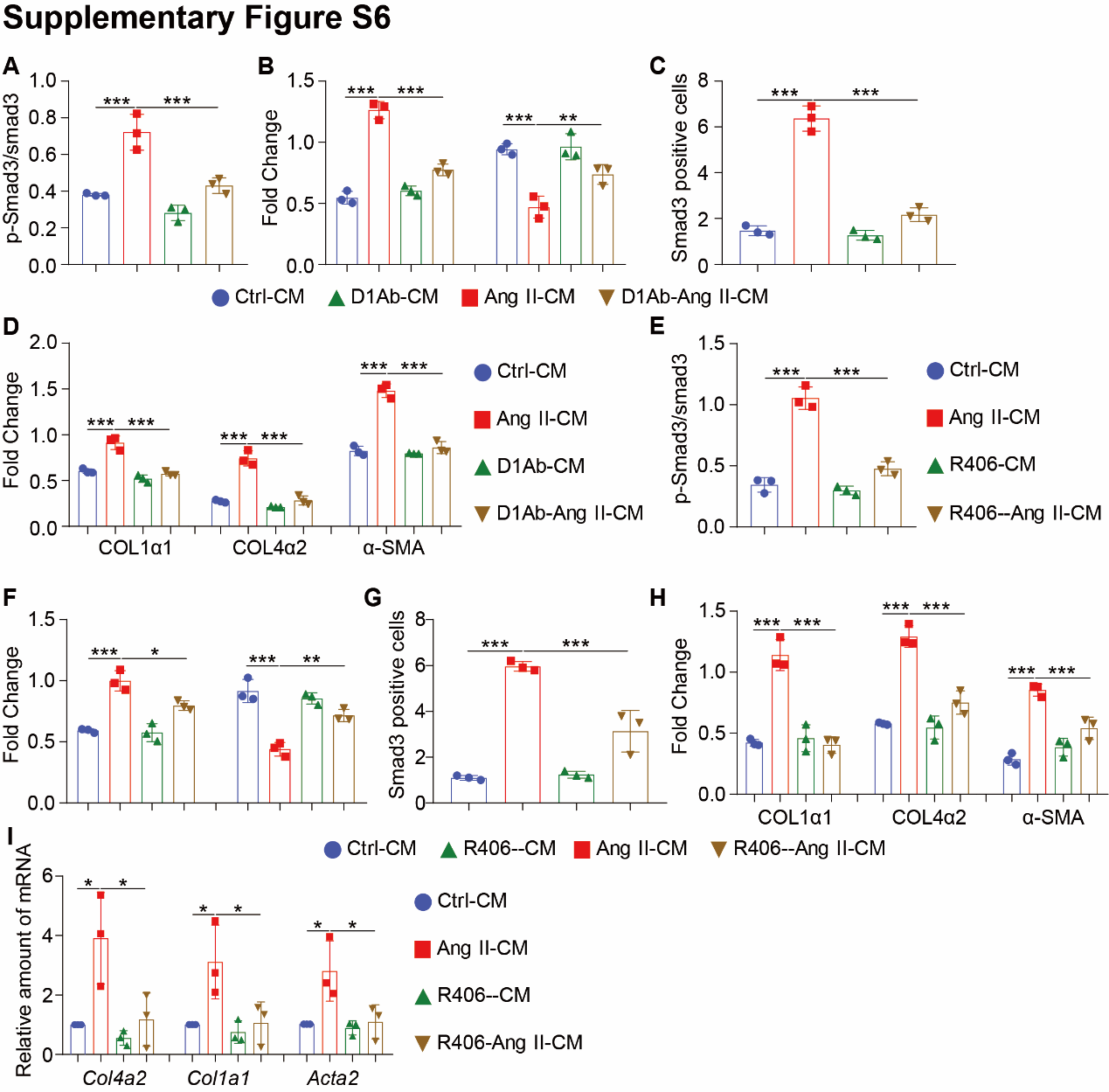


**Supplement Figure S6: Supernatant from Dectin-1/Syk inhibited macrophage alleviated renal fibrosis in vitro. Related to Figure 6.**

(A) Densitometric quantification of immunoblots in in Figure 6A showing p-Smad3: Smad3. [n=3, one-way ANOVA followed by Tukey post-hoc tests, number of comparisons=6, *P<0.05, **P<0.01, and ***P<0.001].

(B) Densitometric quantification of immunoblots in in Figure 6B showing Smad3: LaminB and Smad3: GAPDH. [n=3, one-way ANOVA followed by Tukey post-hoc tests, number of comparisons=6, *P<0.05, **P<0.01, and ***P<0.001].

(C) The quantification of Smad3 nuclear translocation in Figure 6C. [n=3, one-way ANOVA followed by Tukey post-hoc tests, number of comparisons=6, *P<0.05, **P<0.01, and ***P<0.001].

(D) Densitometric quantification of immunoblots in Figure 6D showing COL1α1,: GAPDH, COL4α2: GAPDH and and α-SMA: GAPDH [n=3, one-way ANOVA followed by Tukey post-hoc tests, number of comparisons=6, *P<0.05, **P<0.01, and ***P<0.001].

(E) Densitometric quantification of immunoblots in in Figure 6F showing p-Smad3: Smad3. [n=3, one-way ANOVA followed by Tukey post-hoc tests, number of comparisons=6, *P<0.05, **P<0.01, and ***P<0.001].

(F) Densitometric quantification of immunoblots in in Figure 6G showing Smad3: LaminB and Smad3: GAPDH. [n=3, one-way ANOVA followed by Tukey post-hoc tests, number of comparisons=6, *P<0.05, **P<0.01, and ***P<0.001].

(G) The quantification of Smad3 nuclear translocation in Figure 6H. [n=3, one-way ANOVA followed by Tukey post-hoc tests, number of comparisons=6, *P<0.05, **P<0.01, and ***P<0.001].

(H) Densitometric quantification of immunoblots in Figure 6I showing COL1α1,: GAPDH, COL4α2: GAPDH and and α-SMA: GAPDH [n=3, one-way ANOVA followed by Tukey post-hoc tests, number of comparisons=6, *P<0.05, **P<0.01, and ***P<0.001].

(I) Real-time qPCR showing mRNA levels of *Col4a2, Col1a1* and *Acta2* of each group in SV40 cells. [n=3, one-way ANOVA followed by Tukey post-hoc tests, number of comparisons=6, *P<0.05, **P<0.01, and ***P<0.001].ssss
